# A case of acute abdomen caused by spontaneous rupture of a splenic abscess secondary to cancer of the splenic flexure

**DOI:** 10.1093/jscr/rjab048

**Published:** 2021-04-13

**Authors:** Efstathios T Pavlidis, Eirini K Martzivanou, Nikolaos G Symeonidis, Kyriakos K Psarras, Alexandra G Marneri, Kalliopi E Stavrati, Theodoros E Pavlidis

**Affiliations:** Aristotle University of Thessaloniki, School of Medicine, Second Surgical Propedeutic Department, Hippocration Hospital, Thessaloniki, Greece; Aristotle University of Thessaloniki, School of Medicine, Second Surgical Propedeutic Department, Hippocration Hospital, Thessaloniki, Greece; Aristotle University of Thessaloniki, School of Medicine, Second Surgical Propedeutic Department, Hippocration Hospital, Thessaloniki, Greece; Aristotle University of Thessaloniki, School of Medicine, Second Surgical Propedeutic Department, Hippocration Hospital, Thessaloniki, Greece; Aristotle University of Thessaloniki, School of Medicine, Second Surgical Propedeutic Department, Hippocration Hospital, Thessaloniki, Greece; Aristotle University of Thessaloniki, School of Medicine, Second Surgical Propedeutic Department, Hippocration Hospital, Thessaloniki, Greece; Aristotle University of Thessaloniki, School of Medicine, Second Surgical Propedeutic Department, Hippocration Hospital, Thessaloniki, Greece

## Abstract

Splenic abscesses are rare, difficult to diagnose, difficult to treat and usually appear in immunosuppressed patients. We present the case of a 64-year-old patient with left pleuritic chest pain, anorexia and fever with rigors diagnosed with splenic abscess due to splenic flexure colon cancer. The abscess spontaneously ruptured and the patient was operated on for acute abdomen. Splenectomy and Hartmann’s hemicolectomy were performed. The patient was discharged from the hospital and referred to the oncologic department. Continuous spread of infection and especially initiating from a cancer lesion is a usual mechanism of splenic abscess formation. Although computed tomography-guided percutaneous drainage is the treatment of choice, an exploratory laparotomy was necessary in this case because of the rupture of the abscess. It is important for the clinicians to include splenic abscesses and their complications in the differential diagnosis of acute abdomen.

## INTRODUCTION

A patient with a rare spontaneous rupture of a splenic abscess causing acute abdomen as the first indication of splenic flexure cancer was managed successfully by emergency excision. Splenic abscesses are extremely rare entities, with a reported incidence in 0.14–0.70% autopsies [1, 2]. Common symptoms of the condition include fever, abdominal pain, nausea and vomiting have been described as the most common symptoms [3]; furthermore, the associated risk factors include human immunodeficiency virus disease, intravenous drug abuse, infectious endocarditis and transplantation. Among the five recognized etiologies, splenic abscesses are most commonly caused by the hematogenous spread of infection [4]; other causes include hemoglobin pathologies, chemotherapy, trauma, and contiguous spread of infection [3]. Furthermore, computed tomography (CT) is the preferred diagnostic modality.

**Figure 1:**
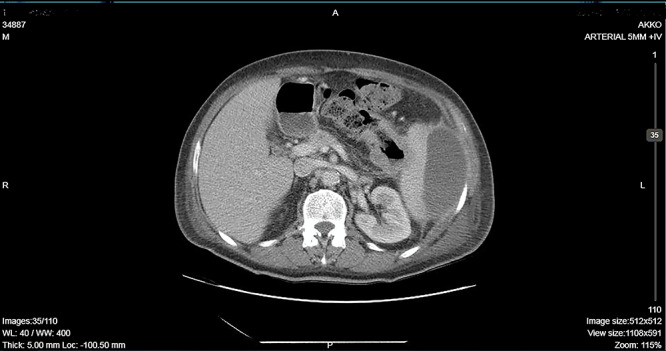
CT showing descending colon wall thickening in contact with the spleen and the tail of the pancreas and a subcapsular splenic abscess as well as splenic vein thrombosis.

**Figure 2:**
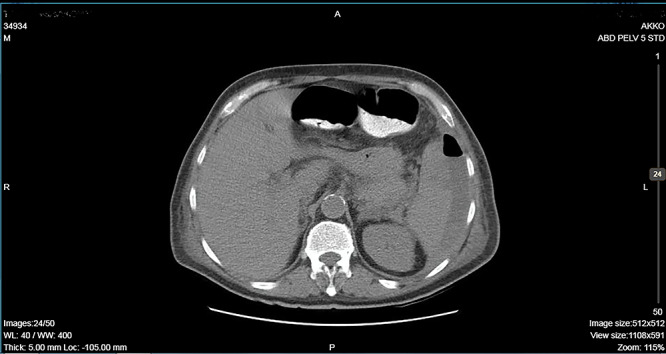
CT showing an important decrease of the abscess due to its rupture toward the peritoneal cavity and presence of air in the abdominal cavity.

## CASE REPORT

A 64-year-old man presented to the emergency department with left pleuritic chest pain, anorexia and fever with rigors. His vital signs included a pulse of 130 beats/min, blood pressure of 108/51 mmHg, respiratory rate of 18 breaths/min, oxygen saturation of 95% and body temperature of 36.1°C. He had no relevant medical history. The patient was admitted to the hospital with the diagnosis of pneumonia based on his chest X-ray findings. The patient remained hemodynamically stable through the next day but developed a temperature of 39.2°C. A CT scan revealed a tumor of the splenic flexure that had invaded the spleen and pancreas via the transcoelomic route and created a subcapsular splenic abscess (Fig. 1). The CT scan also revealed splenic vein thrombosis located proximal to the tail of the pancreas. CT-guided percutaneous drainage of the abscess was scheduled for the following day. The patient’s clinical condition suddenly deteriorated and he became hemodynamically unstable. Abdominal distention with diffuse tenderness was identified via clinical examination, and laboratory tests revealed leukocytosis with a white blood cell count of 16.800/mm and anemia, with a hematocrit of 28.5%. An emergent CT scan revealed intraperitoneal rupture of the splenic abscess (Fig. 2). Intraoperative findings of an urgent surgical intervention included severe, diffuse purulent peritonitis with a large amount of pus mixed with blood clots in the peritoneal cavity that required cautious cleaning and thorough lavage. Furthermore, a large, hard, immovable tumor was identified in the splenic flexure of the colon. The tumor was tightly adherent to the spleen; we also noted the presence of intense inflammation and local fibrosis that extended to the surrounding tissues. En-block splenectomy and a wide left colectomy were performed followed by closure of the distant colon remnant using a linear stapler; furthermore, we also created an ostomy of the proximal colon remnant, similar to Hartmann’s procedure and drained the abdominal cavity. Transfusion of 600 ml of red blood cells was required. Extubation and postoperative recovery were uneventful, and the patient’s clinical status and parameters remained stable. Antibiotic treatment included meropenem 2 g thrice daily, metronidazole 500 mg thrice daily and amikasin 1 g once daily. The postoperative course was uneventful and the patient was discharged on postoperative day 12. Histopathological examination of the resected specimen revealed a 6 cm diameter perforated tumor adherent to an 18 × 13 × 6 cm spleen and a moderately differentiated grade 2 adenocarcinoma with infiltration to the pericolic fat. The resection margins were free of infiltration as were all of the 19 resected lymph nodes. A metastatic tumor, 2 cm in diameter, was identified in the spleen portal. The TNM stage was T3N0M1a (stage IVA disease), and the patient was referred to the Oncologic Department for adjuvant chemotherapy. He was administered eight cycles of Capecitabine that is changed into 5-fluorouracile + Oxaliplatin along with the necessary follow-up care. The patient has been disease-free for 2 years after the treatment.

## DISCUSSION

Splenic abscesses are uncommon and are usually arise from the systemic spread of infection. Splenic flexure cancer is also uncommon, accounting for 4% of all colon cancers, and it has a relatively poor prognosis [5]. Both pathologies were observed in our patient, with splenic abscess formation and rupture following direct invasion of the spleen by the tumor in the splenic flexure. Awotar *et al*. [6] described a similar case and proposed a possible etiological mechanism, following the creation of a splenocolic fistula, the gut flora consequently passes into the spleen and result in the formation of an abscess. The patient was a 59-year-old man with left quadrant pain and low-grade fever as the only symptoms; surgical intervention was administered following the diagnosis of a primary splenic abscess. Barrón-Reyes *et al*. reported a case of splenic abscess rupture in an immunocompromised individual. It was diagnosed during an exploratory laparotomy performed after pneumoperitoneum was seen on simple radiographic exams. There were no signs of malignancy [7].

The relative high mortality rate of splenic abscesses can be attributed to the fact that they are frequently misdiagnosed. CT-guided percutaneous drainage is the preferred treatment for uncomplicated splenic abscesses, considering that it ensures the preservation of the spleen and its immunological functions. Post-splenectomy infections are difficult to treat and have high mortality. Surgical management is preferred in complicated cases with intrabdominal rupture of the abscess. In our patient, it was necessary to thoroughly clean the peritoneal cavity and to perform postoperative drainage.

In conclusion, splenic abscess is a rare condition that is associated with high mortality because it is often misdiagnosed. Direct invasion of the spleen by cancer of the splenic flexure is not a common occurrence and its spontaneous rupture is even rarer. Furthermore, this pathology is difficult to diagnose owing to the absence of specific symptoms. It is important for surgeons to include splenic abscess in the differential diagnosis of acute abdomen.

## CONFLICT OF INTEREST STATEMENT

None declared.

## FUNDING

None.
